# Outcomes of closed interlocking intramedullary nailing of the tibia without fluoroscopy in resource-limited settings: experience from two hospitals in Cameroon

**DOI:** 10.11604/pamj.2021.38.87.26599

**Published:** 2021-01-26

**Authors:** Freddy Mertens Bombah, Guemse Emmanuel Mohamadou, Théophile Nana, Boukar Yannick Ekani, Celestin Danwang, Marc Leroy Guifo

**Affiliations:** 1 Department of Surgery and Specialties, Faculty of Medicine and Pharmaceutical Sciences, University of Douala, Douala, Cameroon,; 2 Department of Surgery and Specialties, Faculty of Medicine and Biomedical Sciences, University of Yaoundé I, Yaoundé, Cameroon,; 3 Department of Surgery and Specialties, Faculty of Medicine and Pharmaceutical Sciences, University of Buea, Cameroon

**Keywords:** Tibia, closed interlocking intramedullary nailing, fluoroscopy

## Abstract

**Introduction:**

closed static interlocking nailing with c-arm guidance is the standard procedure for the treatment of closed diaphyseal leg fractures. It is still very difficult to carry out such procedures in a low-income setting because of few or absent image intensifiers (c-arm) despite the necessity. The aim of this study was to describe the outcomes of patients with tibial fractures treated with closed interlocking intramedullary nails without c-arm guidance in a Cameroonian population.

**Methods:**

this was a prospective study including adult patients treated for tibial fractures without a c-arm in two regional hospitals.

**Results:**

finally, 22 patients were included. The mean age was 34 ± 12.6 years with a male predominance (16 males and 6 females). Ninety percent of the fracture lines were simple or with a wedge fragment grade 42A or 42B respectively according to the AO classification. The mean surgery time was 1 hour 26 ± 34 minutes. The various aspects evaluated were the nail entry point which was good in 19 (86.4%) cases; proper nail driving which was considered good in 15 (68%) cases; the distal locking which was missed in 6 (27.3%) cases. Bone consolidation was obtained in an average of 4 ± 1.2 months in all 22 cases.

**Conclusion:**

in resource constraints settings where c-arm are not always available, closed interlocked nail of tibia without c-arm guidance still gives overall good results. Nevertheless, there is a need to improve equipment in sub-Saharan African hospitals to make trauma surgery with c-arm a gold standard as currently recommended.

## Introduction

Lower limb fractures are among the more common types of fractures in sub-Saharan African [1, 2]. In Cameroon, fracture of the tibia is common among young adults, and is challenging for surgical teams because of the scarcity of resources to provide and optimally treatment to those patients, respecting international guidelines [3]. Indeed, closed interlocked nail with c-arm guidance is the gold-standard for the treatment of long bone fractures of the lower limbs [4]. Therapeutic indications for surgical treatment of long bone fractures have evolved within the years, starting from the original works done by Kuntcsher to the static locking technic developed by French teams [5, 6], using c-arm control. Static nailing should be done using specific ancillary devices which are not always available in low-income settings. Closed reduction and static locked nailing appear therefore as a reliable alternative and have widely been study in other sub-Saharan African countries like Nigeria [7, 8] and Uganda [9].

Four essential aspects have been reported to be important in the achievement of a successful nailing without c- arm: the nail entry point, the progress of the guide wire through the distal segment, fracture reduction, and locking screws insertion. In Cameroon, a similar study was done but with and open reduction of the fracture [10]. This study reported the difficulties and outcomes of patients with closed interlocked nailing of tibia fractures without c- arm guidance performs in two hospitals in Cameroon.

## Methods

**Study setting:** this was a prospective study carried out in two hospitals in Cameroon: the regional hospital Ebolowa, and Ad-lucem hospital Bonamoussadi in Douala between October 2016 and October 2018.

**Study population:** patients included were adults aged more than 18 years with closed fracture of the tibia, going throw a static locked nails procedure without the use of c-arm and giving their consent to be included in the study. Patients with multiple fractures or with open tibia fractures were excluded.

**Data collection:** patients were identified from consultation/hospitalization registers, X-ray and operating reports. Their files were consulted to complete the preconceived data collection form. The following information was recorded: sociodemographic (name, surname, age, gender, profession), clinique (Glasgow coma scale, vital parameters, presence of comorbidities, all lesions and their characteristics), and the outcomes.

**The outcomes were:** the quality of the nailing (position of the nail and the placement of the locking screws) which was assessed using 3 criteria [10]; the entry point: slightly medial behind the tibial tuberosity; the insertion of the nail: nail drive from the proximal fragment to the distal fragment with reduction of the fracture site; the insertion of locking screws: passage of the locking screw through the eyelet of the nail; bone consolidation which was define by presence of a homogeneous bone callus on the X-ray; functional outcome, which was classified as excellent, good, fair, or poor. The quality classification was made based on criteria of Kalstrom and Olerud: walking, pain, joint integrity, morphology [11].

Pre-operative planning was systematically done for all the cases using their X-rays. The site of fractures, the line of fracture, displacements, and associated lesions were studied on X-rays. The dimensions of the cortices and medullary canal were noted. These helped to evaluate the size of the nail and length of screws to be used. All the patients had pre-operative laboratory works, and pre-anesthetic evaluation. The specific material used (ancillary device) was from Sharma orthopaedics.

**Figure 1:**
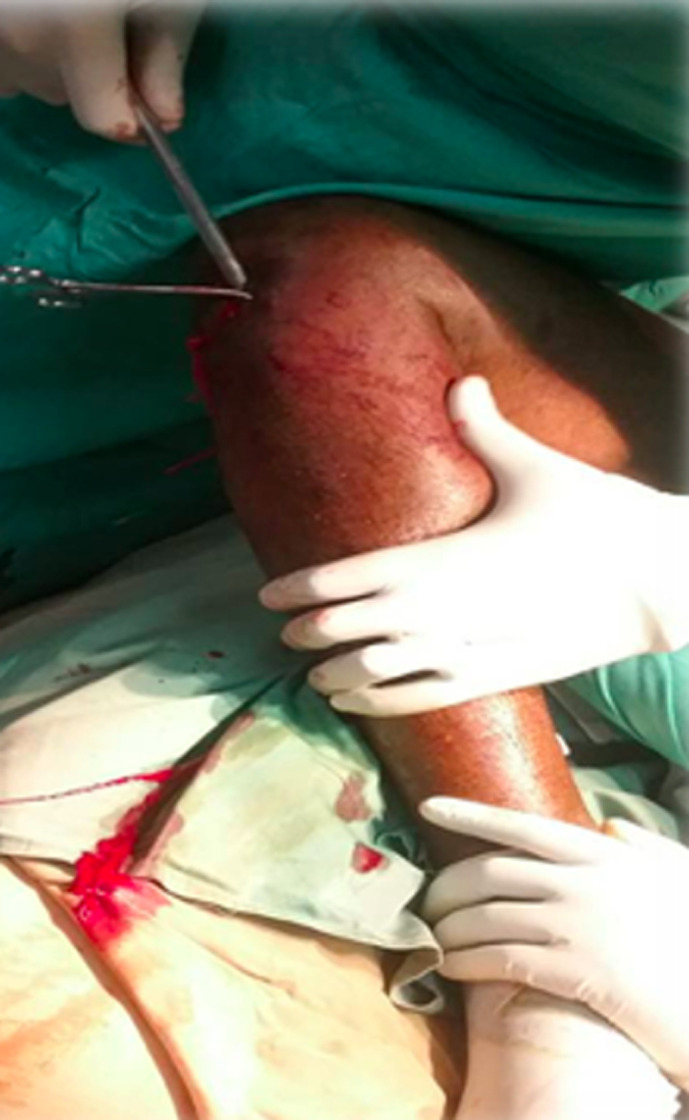
maintenance of reduction of the fracture hole and reaming with a manual rigid reamer

**Figure 2:**
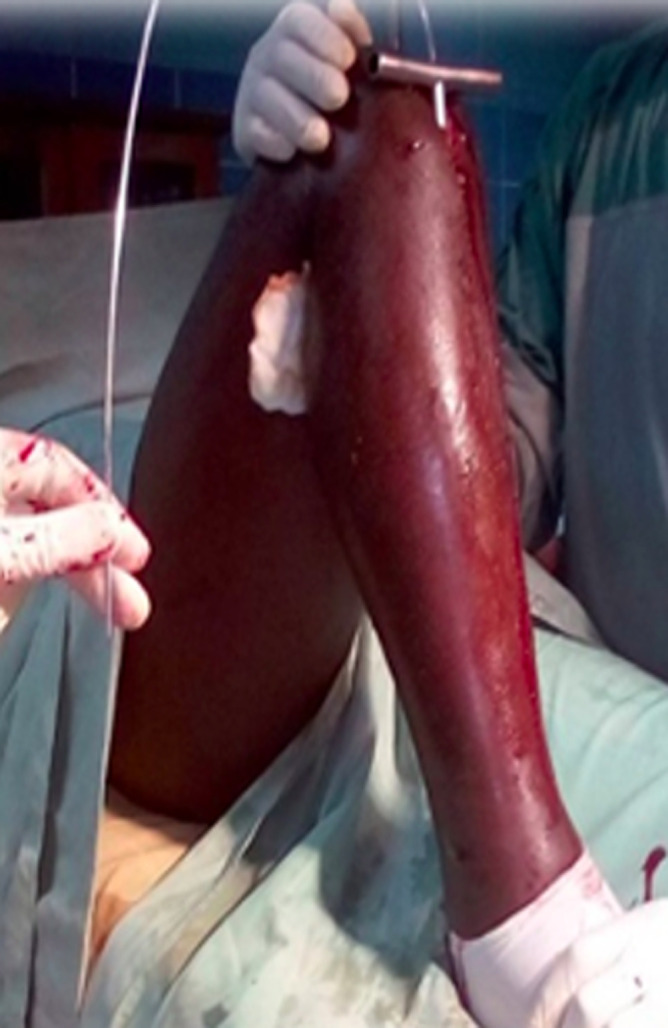
guide put in place through the manual rigid reamer

The patients were all placed supine in bed with the knee of the injured leg flexed to 120 degrees. A direct infrapatellar approach was done exposing the anterior tibia tuberosity. Closed reduction of the fracture was done with external maneuvers to obtain a continuity of the tibial crest and the medial aspect of the tibia. The reduction obtained was maintained by the assistant (Figure 1). The progress of the guide wire was done in the canal of the proximal and distal segments (Figure 2) through the rigid reamer inserted first. After manual reaming, the nail was then inserted through the guide. Distal locking was done first before proximal locking. Nail insertion was termed good when the proximal end was lower than the tibia plateau and just above the anterior tibia tuberosity. A diastasis was noted if the fragments were more than 0.5 cm apart. The locking was termed not good if the screws didn´t pass through the holes of the nail.

The functional outcome was characterized as excellent, good, fair, or poor [11]. The quality classification was made based on criteria of Kalstrom and Olerud: walking, pain, joint integrity, morphology.

**Data analysis:** data were analysed with Epi info 3.5.1 software. Because of the sample size greater than 20, parametric tests were used assuming the normality of the distribution of outcomes variables (central limited theorem). Thus, a t-test was used to compare the mean within two categories and a one-way ANOVA, between more than two categories. Chi2 or Fisher exact test were used when appropriated to compare proportions. Patients lost to follow-up were excluded from the final analysis and a p-value > 5% was considered statistically significant.

**Ethical considerations:** prior to inclusion in the study, and inform consent was obtained from all patients.

## Results

**General characteristics:** overall, 22 patients were included in the study. Concerning the gender, 16 (72,7%) were male and 6 (27,3%) females. The number of diaphyseal tibia fractures was 22 (one per patient). The mean age was 34 ± 12.6 years and the most common cause of fracture was road traffic crash (86.3% of the 22 cases). Other causes were falls (2 cases, 9%), and a case (4 %) of assault. The fracture was classified AO 42A (54.5%, n=12) and 42B (36.4%, n=8), (Table 1). The right leg was mostly involved in (14 cases, 63.6%). The site of fractures was mid-diaphyseal in 82% (n=18) of cases. The mean duration of surgery was 1h 26 ± 34 minutes.

**Table 1:** summary table of patients treated by static interlocking intramedullary nailing of the tibia without fluoroscopy

Patients	Age	Sex	Location of the fracture	AO Classification	Complication	Results
1	24	M	Left	42 A1	**-**	Excellent
2	34	F	Right	42 A2	**-**	Excellent
3	17	M	Left	42 C1	**-**	Acceptable
4	30	M	Right	42 A2	**-**	Good
5	44	M	Right	42 A3	**-**	Excellent
6	51	F	Right	42 B2	**-**	Good
7	38	M	Left	42 A1	**-**	Excellent
8	20	F	Left	42 B2	**-**	Good
9	64	M	Right	42 A2	delayed union	Acceptable
10	48	M	Right	42 B1	**-**	Excellent
11	34	M	Left	42 B2	**-**	Excellent
12	24	F	Right	42 A 2	**-**	Good
13	40	M	Right	42 B2	**-**	Good
14	27	M	Right	42 A1	**-**	Good
15	21	M	Left	42 C2	**-**	Bad
16	28	M	Left	42 A3	**-**	Acceptable
17	59	M	Left	42 B2	**-**	Excellent
18	25	F	Right	42 A2	Vicious callus	Excellent
19	25	M	Right	42 A1	**-**	Excellent
20	27	M	Right	42 B2	**-**	Excellent
21	34	M	Right	42 A2	**-**	Excellent
22	34	F	Right	42 B2	**-**	Acceptable

**Outcomes:** no complications were recorded during the surgery. The position of the nail and the placement of the locking screws were evaluated using 3 criteria: the entry point, the insertion of the nail, and the insertion of locking screws. The entry point was good in 20 (90.9%) cases. The nail insertion was good in 18 (81.8%) cases. The distal locking was missed in 5 (22.7%) cases. Proximal locking was good in all the cases. There were 3 (14 %) cases of diastasis which were later managed by dynamizing the nail within 6 weeks of surgery. Consolidation was obtained in all cases during a mean period of 4 ± 1.2 months. There was a case of delayed union and no cases of non-union nor infection. All the cases had full weight bearing without crutches after 6 weeks. The clinical outcome was excellent in 11 (50%) cases, good in 5 (27.2%) cases, fair in 4 (18.3%) cases and poor in 1 (4.5%).

## Discussion

The current study found and overall good outcomes of patients after closed static interlocking nailing without c-arm in two Cameroonian hospital. Our findings are not the first to point to good outcome of patients after this procedure. Indeed, static interlocking nailing without the use of image intensifier has been study previously in others sub-Saharan Africa [7-9]. All of them used Surgical Implant Generation Network (SIGN). Guifo and collegues in Cameroon in 2016, studied this technic with titanium nails of 10mm diamenter (Jiangsu Jinlu group Medical DEVICE CO). None of the above-mentioned studies used closed reduction and interlocking nailing without c-arm guidance. In our case series, we treated patients by closed reduction and static interlocked nailing without image intensifier using nails of 9- and 10-mm diameters. (Sharma orthopedic group).

Bone consolidation occurred in all the cases, and we had a functional outcome that was considered excellent or good in 64% cases of femur and tibia nailing. However, we had a case of delayed union and a case of malunion which didn´t affect patients´ wellbeing. Our technic is advantageous because of absence of exposure to radiations. We evaluated 4 points which are objectives of interlocking nails done without image guidance according to Guifo *et al*. [10]; the entry point of the nail, the passage of the guide wire into the distal segment, fracture reduction, the insertion of the locking screws and presence of contact between bone fragments. The choice of the entry point is very important in in the placement of nails [4]. A more lateral or more medial entry point will lead to a mispositioning of the guide wire and a varus or valgus fixation. A very low entry point may lead to protrusion of the nail to the skin [12]. The entry point was good in 90.9% of cases. This result is similar to that obtained by Guifo *et al*. who 93% for tibia. The use of c-arm control doesn´t guarantee a successful nailing [13]. The passage of the guide wire through the distal fracture segment was done without opening the fracture site and without the use of c-arm control. This was achieved by external manipulation of both segments so that the guide could pass and also with the help of a rigid reamer. Care was taken not to further injure soft tissue.

The 3 cases of diastasis of the fracture fragments were managed by early dynamization of the nails. Guifo *et al*. also had 3 cases of diastasis. Dynamization was initially recommended by Kempf *et al*. [14]. Removal of locking screws was done early in cases where the patients complain of discomfort [15]. Other authors studied interlocking nails without the use of c-arm [7-9] using ancillary devices that target the holes in the nails for the passage of the locking screws. Insertion of the proximal screws is most often easy; however, distal locking remains very difficult in 100% of cases because of divers´ reasons amongst which are; poor entry point, and poor assembling of ancillary device. Distal locking was done before proximal locking and was missed in 5 cases (22.7%). This result is similar to those obtained by Guifo *et al*. [10] who had 3 cases of missed distal locking of tibia fractures (Table 2). We considered that distal locking was successful when rotational movements of the nail in the medullary canal were impossible. In the 5 cases where distal locking was missed, the distal screws acted therefore as blocking screws by maintaining the alignment of the nail and adding stability according to Mugundhan Moongilpatti S *et al*. [16].

**Table 2:** evaluation of nail positioning and quality of locking

Parameters	Assessment result	
	M.L. Guifo et al	Our
Nail entry point	Good [13 (93%)]	Good [19 (86,4%)]
Nail driving	Good [11 (78,5%)]	Good [16 (72,7%)]
Diastasis	3 (21,5%)	3 (13,6%)
Distal locking	3 (21,5%)	5 (22,7%)

The height of the nail was good in 18 cases (81.8%). This finding is similar to that obtained by Guifo *et al*. who 78.5% good results. Stainless steel nails of 9 or 10mm diameter were used after adequate reaming in our study while other studies report use of titanium nails with or without reaming [17, 18] (Table 2). A good indication for surgery is primordial for doing this technique. In our study, 91% of cases were 42A and 42B according to AO. Type 42C fractures and supra metaphyseal fractures had poor results and developed more complications subsequently. The functional results were excellent or good in 77.2% of cases on the scale proposed by Kalström *et al*. The main limit of our study is the small sample size. Nevertheless, this preliminary study could be used to design studies on the practice of locked intramedullary nailing without image intensifiers in countries with limited resources.

## Conclusion

In resource constraints settings where c-arm are not always available, closed interlocked nail of tibia without c-arm guidance still gives overall good results. Nevertheless, there is a need to improve equipment in sub-Saharan African hospitals to make trauma surgery with c-arm a gold standard as currently recommended.

### What is known about this topic

- Closed static interlocking nailing with c-arm guidance is the gold-standard procedure for the treatment of closed diaphyseal leg fractures;
- It is still very difficult to performed interlocking nailing with c-arm in a low-income setting because of few or absent image intensifiers;
- Closed interlocking nailing without the use of c-arm has shown good results in some studies conducted in sub-Saharan Africa and have been proposed as an alternative to the gold-standard in resources constraints settings.

### What this study adds

- The current study is among the first multicentre study perform on closed interlocking nailing without the use of c-arm in Cameroon;
- Our findings suggested overall good outcomes of patients after closed static interlocking nailing without c-arm in two Cameroonian hospitals.
